# Metal-organic framework-derived metal oxide nanoparticles@reduced graphene oxide composites as cathode materials for rechargeable aluminium-ion batteries

**DOI:** 10.1038/s41598-019-50156-6

**Published:** 2019-09-24

**Authors:** Kaiqiang Zhang, Tae Hyung Lee, Joo Hwan Cha, Ho Won Jang, Ji-Won Choi, Morteza Mahmoudi, Mohammadreza Shokouhimehr

**Affiliations:** 1 Department of Materials Science and Engineering, Research Institute of Advanced Materials, Seoul National University, Seoul, 08826 Republic of Korea; 2 Electronic Materials Center, Korea Institute of Science and Technology (KIST), Seoul, 136-791 Republic of Korea; 3 Innovative Enterprise Cooperation Center, Korea Institute of Science and Technology (KIST), Seoul, Republic of Korea; 4 Precision Health Program, Michigan State University, East Lansing, MI 48823 USA

**Keywords:** Batteries, Batteries

## Abstract

The use of metal oxides as electrode materials has seen great success in lithium-ion batteries. However, this type of electrode materials has been regarded as an improper option for rechargeable aluminium-ion batteries (AIBs) in comparison with sulfides and selenides, and has, thus, been nearly abandoned. Here, we demonstrate the suitability of metal oxides as cathode materials of AIBs, exhibiting high electrochemical activities toward Al-ion storage. We designed economical metal-oxide cathodes (Co_3_O_4_@reduced graphene oxide (rGO), Fe_2_O_3_@rGO, and CoFe_2_O_4_@rGO) for AIBs. The Co_3_O_4_@rGO displayed superior electrochemical properties, regarding both capacity and lifespan, to the current state-of-the-art cathode material reported by scientific literature. Furthermore, the CoFe_2_O_4_@rGO exhibits rational electrochemical capacities and an extremely stable charge/discharge process with an excellent Coulombic efficiency of 99.6%. The proposed study expects to stimulate researchers to focus on the overlooked metal oxides as competitive cathode materials for high performance AIBs.

## Introduction

With the ever-growing environmental problems and ever-declining storage amounts of fossil energy resources, methods of integrating green and sustainable energy resources (such as solar, tidal, and wind energies) into an electric grid have been intensively researched^1–6^. However, a common issue for natural energy resources is the intermittency, which requires the employment of intermediate high-performing and cost-efficient energy storage devices^7^. Aluminium-ion batteries (AIBs), as post-lithium-ion batteries, are regarded as potential candidates for the next generation of electric energy storage, owing to the uniquely high charge density (Al^3+^) and rich substantial reserves in the earth (~8000 ppm, <2.5 $ kg^−1^)^8^. After the significant advance on AIBs made by Dai’s group^9^, an intensive study on AIBs has been performed, focusing on the development of the cathode materials as an Al metal can be used directly as an anode and the employment of ionic-liquid electrolyte ([EMIM]Cl/AlCl_3_) enables stable Al stripping and plating in AIBs. Reliable cathode materials for AIBs which can tolerate the high charge density (Al^3+^) and large ionic radius of the inserted ion (AlCl_4_^−^) are important for high-performing AIBs^10^. To meet this requirement, research groups have intensively explored C-based materials (e.g., defect-free graphene, three-dimensional graphene foam, and natural graphite) among other compounds (e.g., sulfides and selenides)^11–14^. Studies on the metal-oxide compounds as cathode materials of AIBs assembled with an ionic-liquid electrolyte are rare due to the high electronegativity of O ions. Metal oxides, such as TiO_2_ and MoO_3_, are initially demonstrated to be decent cathode materials in aqueous AIBs^15,16^. However, further investigation on metal oxides as the cathode materials of non-aqueous AIBs has been overlooked, except a few relevant reports^17,18^ with inferior electrochemical capacities and limited lifespan. Zhang’s group designed CuO microsphere architectures for use as a cathode material of AIBs, demonstrating a rational initial capacity (~250 mAh g^−1^) but a rapid decay (~100 mAh g^−1^ at the 100^th^ cycle) at a charge/discharge current density of 200 mA g^−1^ ^19^. Their findings, thus, inspire us to conduct an in-depth study on this issue.

Metal-organic frameworks (MOFs) have been demonstrated to be a novel class of original or sacrificial materials for multiple applications, including gas absorption, supercapacitors, and batteries, owing to their porous structures and high surface areas^20–22^. The MOFs derived porous and organized electrode materials provide more exposed active sites, short diffusion length, and integrity of the electrode materials during electrochemical reactions. Zhang *et al*. demonstrated porous CuO/Cu_2_O as a high-performance anode material for sodium-ion batteries, exhibiting a reversible capacity of 415 mAh g^−1^ at 50 mA g^−1^ ^23^. Furthermore, Zou *et al*. reported Ni-based MOF-derived NiO/Ni nanocrystals as an advanced anode material of lithium-ion batteries, showing an excellent reversible capacity (1144 mAh g^−1^), cyclability, and rate performance^24^. Recently, Xu *et al*. used an MOF as a precursor to form carbon-encapsulated selenides as superior anode materials for sodium-ion batteries, with a potential electrochemical property of 218 mAh g^−1^ after 500 cycles at a current density of 3,000 mA g^−1^ ^25^. However, reports on MOF-derived metal oxides as cathode materials for AIBs are absent. It is, thus, highly interesting to further study the electrochemical properties of MOF-derived metal oxides as cathode materials for AIBs.

Herein, we demonstrate the advanced electrochemical properties of unit and multiple metal oxides (Co_3_O_4_@reduced graphene oxide (rGO), Fe_2_O_3_@rGO, and CoFe_2_O_3_@rGO) as cathode materials of AIBs, in which the rGO is employed as a wrapping layer to reinforce the material structures. Prussian blue analog (PBA)-type MOF (CoCo(CN)_6_, FeFe(CN)_6_, and FeCo(CN)_6_) are employed as the precursors, which can be constructed by a facile co-precipitation process in an aqueous bath without further hydrothermal treatment. It is found that the synthesized metal-oxide samples exhibit superior electrochemical properties as cathode materials for AIBs.

## Results and Discussion

**Figure 1:**
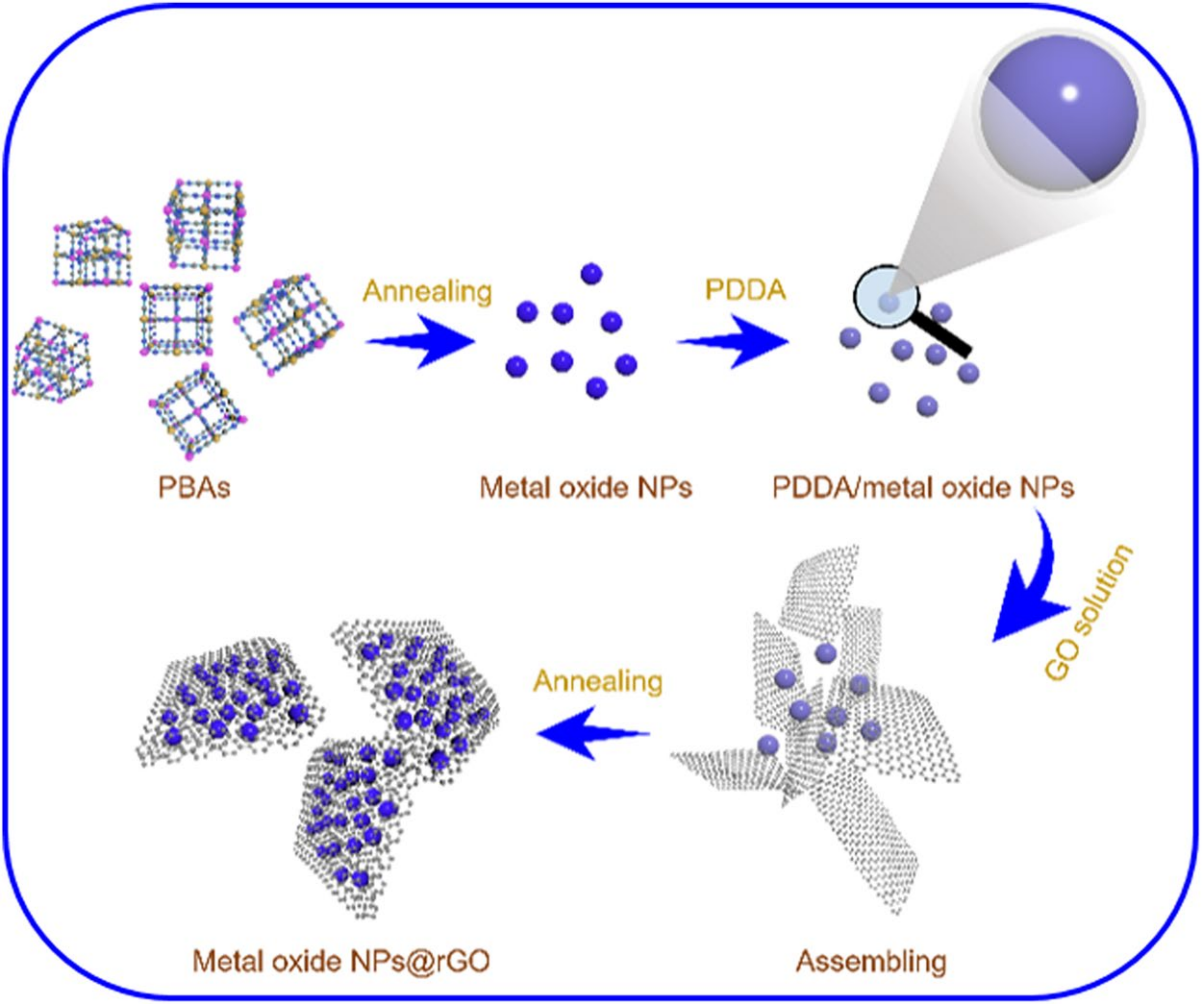
Schematic illustration of the synthesis process of metal-oxide NPs@rGO.

**Figure 2:**
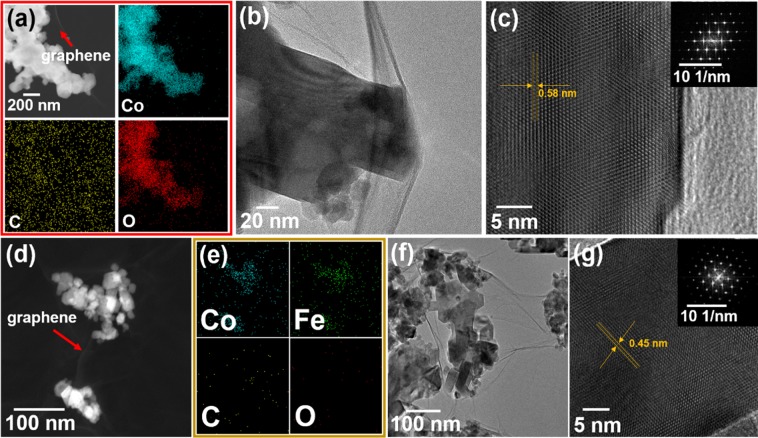
(**a**) STEM and EDX mapping, (**b**) TEM, and (**c**) HRTEM images of Co_3_O_4_@rGO. (**d**) STEM, (**e**) EDX mapping, (**f**) TEM, and (**g**) HRTEM images of CoFe_2_O_4_@rGO.

The synthesis process is illustrated in Fig. 1. The entire process is performed in an aqueous bath and could be easily mass produced. The morphologies of the prepared Co_3_O_4_@rGO, Fe_2_O_3_@rGO, and CoFe_2_O_4_@rGO depicted in Fig. 2 and Supplementary Figs S1–S4 are observed using scanning electron microscopy (SEM) and transmission electron microscopy (TEM). The rGO wrapping layer is clearly observed in the scanning TEM (STEM, Fig. 2a,d), where almost-transparent graphene sheets are exhibited. Such a phenomenon can be reconfirmed by the TEM morphologies (Fig. 2b,f). The consistent elements of the final Co_3_O_4_@rGO, Fe_2_O_3_@rGO and CoFe_2_O@rGO are further qualitatively measured by energy-dispersive X-ray spectroscopy (EDX) (Fig. 2a,e), where the component elements are uniformly distributed over-through each particle.

The same structural and elemental information can also be obtained for Fe_2_O_3_@rGO (Supplementary Fig. S1), which is similar to that of Co_3_O_4_@rGO and CoFe_2_O_4_@rGO, displaying clear pocket-like rGO-wrapped metal-oxide nanoparticles (NPs) (Supplementary Figs S1–S4). The elemental characteristics for the three types of material are further studied with an electron probe microanalyser (EPMA), as shown in Supplementary Fig. S5. The aligned atoms in the high-resolution TEM (HRTEM) and the corresponding fast Fourier transform diffraction patterns depict well-crystallized natures for Co_3_O_4_@rGO, Fe_2_O_3_@rGO, and CoFe_2_O_4_@rGO (Fig. 2c,g and Supplementary Fig. S4). The interplanar spacing of Co_3_O_4_@rGO, Fe_2_O_3_@rGO, and CoFe_2_O_4_@rGO measured using HRTEM are 0.58, 0.37, and 0.45 nm, respectively.

**Figure 3:**
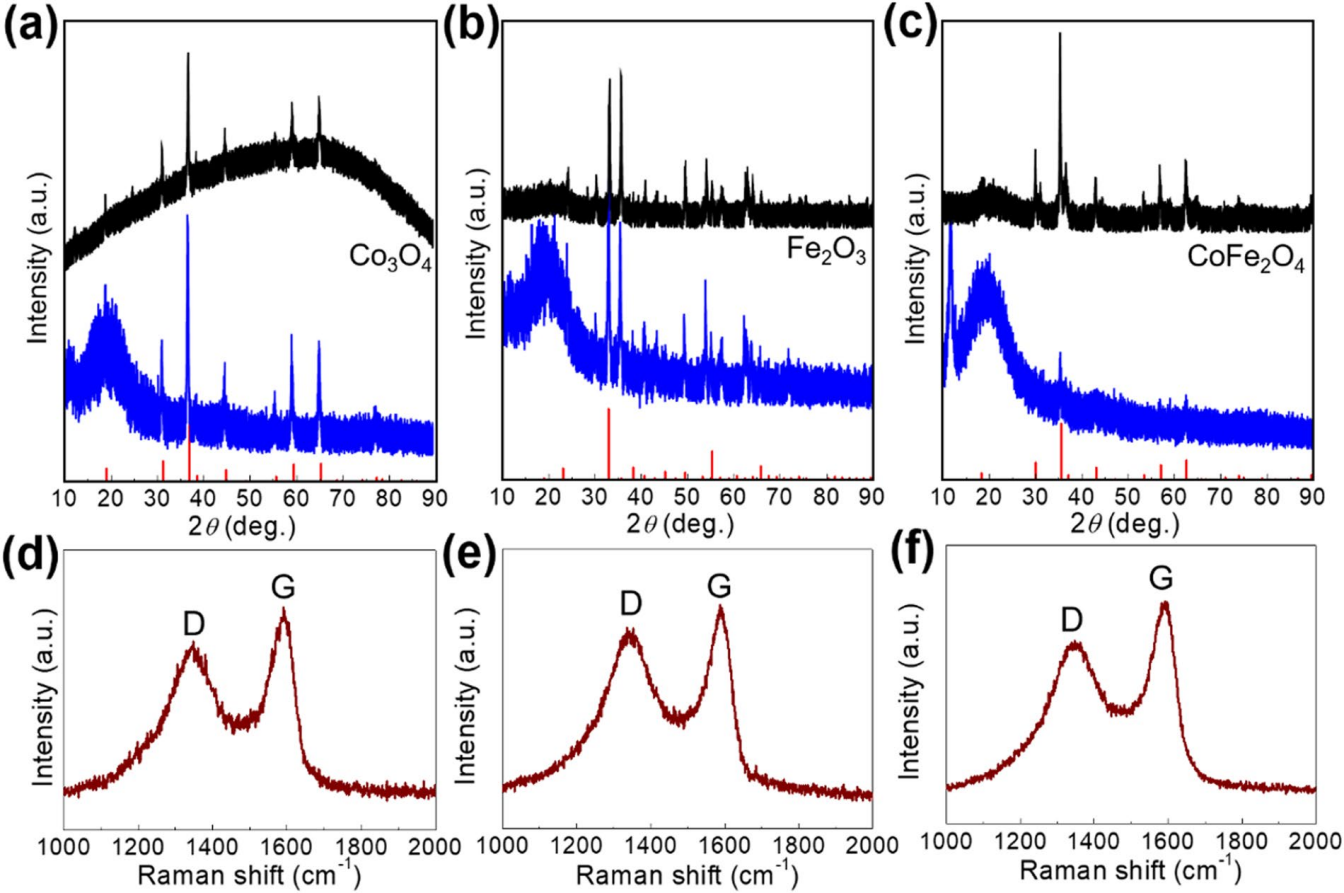
XRD peaks of (**a**) Co_3_O_4_ and Co_3_O_4_@rGO, (**b**) Fe_2_O_3_ and Fe_2_O_3_@rGO, and (**c**) CoFe_2_O_4_ and CoFe_2_O_4_@rGO. Raman spectra of (**d**) Co_3_O_4_@rGO, (**e**) Fe_2_O_3_@rGO, and (**f**) CoFe_2_O_4_@rGO.

**Figure 4:**
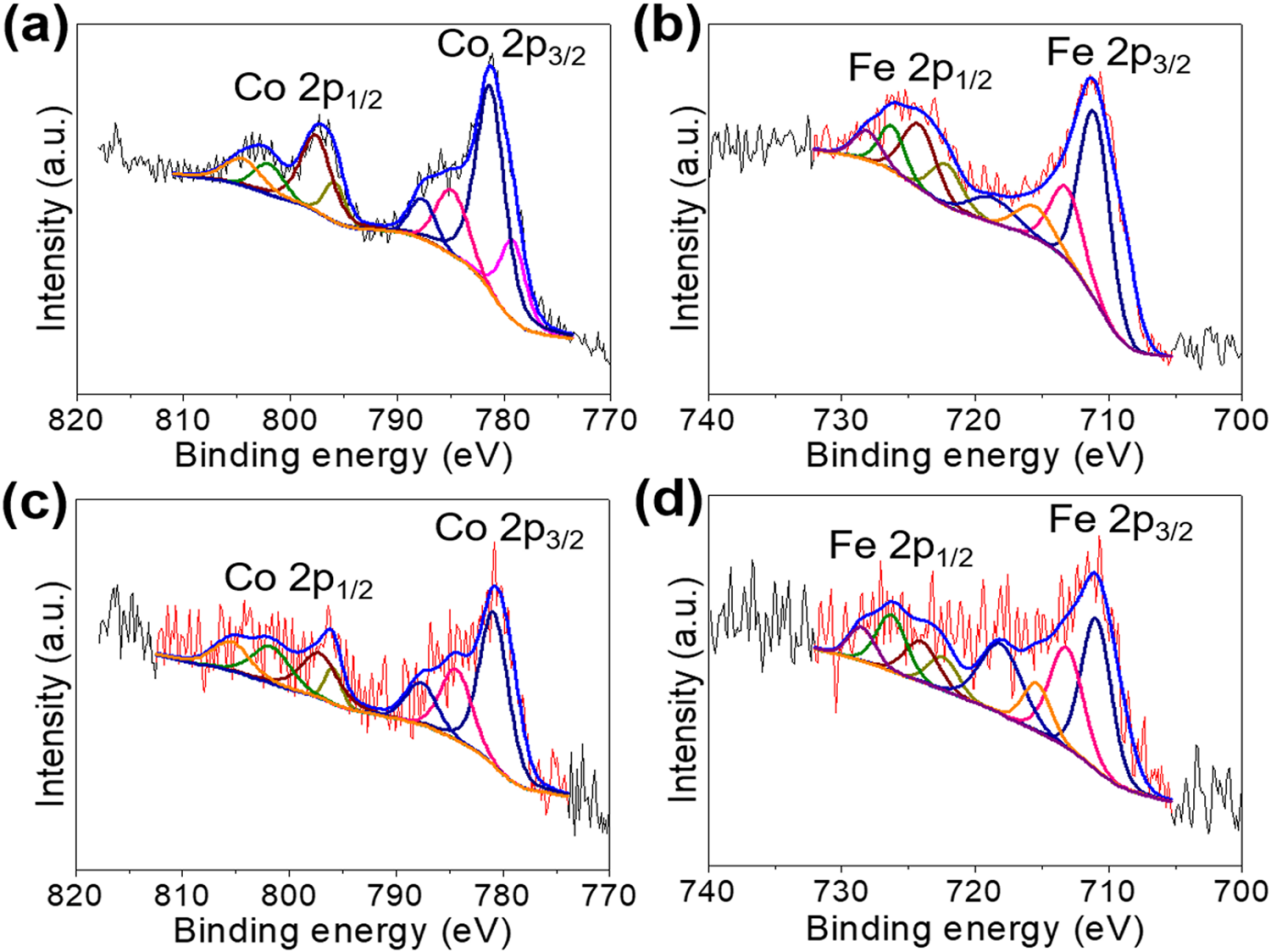
XPS spectra of (**a**) Co 2p of the Co_3_O_4_@rGO, (**b**) Fe 2p of the Fe_2_O_3_@rGO, and (**c**) Co 2p and (**d**) Fe 2p of the CoFe_2_O_4_@rGO.

The structures of Co_3_O_4_@rGO, Fe_2_O_3_@rGO, and CoFe_2_O_4_@rGO are observed via X-ray diffraction (XRD) following a contrastive analysis. After facile heat treatments for the pre-prepared PBAs, Co_3_O_4_, Fe_2_O_3_, and CoFe_2_O_4_ are formed based on the well-indexed diffraction peaks, which can be preserved after the introduction of rGO (Fig. 3). The XRD results of Co_3_O_4_@rGO, Fe_2_O_3_@rGO, and CoFe_2_O_4_@rGO exhibit combined phases of metal-oxide and rGO (humps at ~20°). The introduced protective layer (rGO) is further detected with Raman spectroscopy, as shown in Fig. 3d–f, and the characteristic D and G bands are the same as naked rGO (Supplementary Fig. S6). This indicates well-preserved graphene layers after the self-assembly process in aqueous solutions and annealing processes. The metal-oxide phases in the as-prepared Co_3_O_4_@rGO, Fe_2_O_3_@rGO, and CoFe_2_O_4_@rGO are further detected with X-ray photoelectron spectroscopy (XPS, Fig. 4). Co-existing Co^2+^ and Co^3+^ for Co_3_O_4_@rGO; Fe^3+^ for Fe_2_O_3_@rGO; Co^2+^ and Fe^3+^ for CoFe_2_O_4_@rGO are analysed, which indirectly demonstrate the phase results obtained from the XRD spectra. Meanwhile, a wide survey of the XPS spectra for the three samples (Supplementary Fig. S7) further demonstrates the bonding natures; similar and single deconvoluted diffraction peaks with high intensities are observed for C 1 s, revealing the single-existence state of the rGO in samples. Furthermore, three deconvoluted O 1s peaks (Supplementary Fig. S6c,f,i) correspond to the metal oxide and absorbed water molecules in each sample, with a slight difference in peak intensities.

The thermal stabilities of the as-prepared samples were measured via thermogravimetric analysis (TGA) prior to the electrochemical measurements. Sufficient thermal stabilities until 400 °C for the samples are verified (Supplementary Fig. S8), with less absorbed water species. For the sake of the subsequent electrochemical measurements, the mixture ratios of the three types of materials are measured using X-ray fluorescence (XRF). Besides the qualitative characteristic elemental curves (Supplementary Fig. S9), the metal-oxide NPs/rGO ratios are shown to be 49:51 for Co_3_O_4_@rGO, 62:38 for Fe_2_O_3_@rGO, and 40:60 for CoFe_2_O_4_@rGO (Supplementary Table S1). The obtained results are used as reference values for calculations of the subsequent current densities and specific capacities.

**Figure 5:**
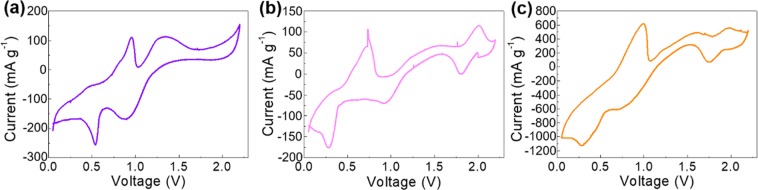
CV curves of (**a**) Co_3_O_4_@rGO, (**b**) Fe_2_O_3_@rGO, and (**c**) CoFe_2_O_4_@rGO from 0.05–2.2 V vs. AlCl_4_^−^/Al.

Electrochemically active voltage ranges of the three cathode materials are diagnosed using cyclic voltammetry (CV) curves from 0.05–2.2 V vs. AlCl_4_^−^/Al, as shown in Fig. 5, where the redox peaks can be clearly observed for each sample. In particular, two pairs of reduction/oxidation peaks at ~0.5/0.8 and 0.9/1.3 V vs. AlCl_4_^−^/Al for Co_3_O_4_@rGO, ~0.25/0.70 and 1.8/2.0 V vs. AlCl_4_^−^/Al for Fe_2_O_3_@rGO, and ~0.3/0.8 and 1.8/2.0 V vs. AlCl_4_^−^/Al for CoFe_2_O_4_@rGO are displayed, demonstrating the typical electrochemical-based charge/discharge processes. Furthermore, stage by stage charge/discharge processes are demonstrated by these multiple peaks. We, thus, discharge the electrodes comprising of the as-prepared products to 0.2 and 0.7 V vs. AlCl_4_^−^/Al for Co_3_O_4_@rGO; 0.1, 0.5, and 1.5 V vs. AlCl_4_^−^/Al for Fe_2_O_3_@rGO; 0.1, 0.5, and 1.5 V vs. AlCl_4_^−^/Al for CoFe_2_O_4_@rGO to reveal the underlying electrochemical reactions. For which, it is necessary to know how much Al species is inserted in the products in each stage. One facile solution is the EDX mapping^7^. By which, the inserted Al species amount increases with discharge going deeper for all products as recorded in Supplementary Tables S2–S4 where the ratios of Co/Al for Co_3_O_4_@rGO, Fe/Al for Fe_2_O_3_@rGO, and Fe(or Co)/Al for CoFe_2_O_4_@rGO are gradually enhanced. Furthermore, the effective electrochemical reaction for each stage can be roughly concluded as follows. The same electrochemical reaction in the anode side can be obtained in different electrochemical stages (Supplementary Eqs S1, S2)^17,18^. In the cathode side, discharge proceeds stage by stage by following the corresponding electrochemical reactions (Supplementary Eqs S3–S10). Al species is inserted into the discharge product formed in the last discharge stage. This multiple electrochemical stages are also demonstrated by the *ex-situ* XRD. The inserted Al species is clearly exhibited in the form of Al oxides due to the exposure in ambient environment (Supplementary Fig. S10).

Theoretical capacities of the products are calculated based on the above discussion. As a result, theoretical capacities of 234 mAh g^−1^ (based on the weight of Co_3_O_4_ and 0.7 mole of Al insertion), 1508 mAh g^−1^ (based on the weight of Fe_2_O_3_ and 1.5 mole of Al insertion), and 444 mAh g^−1^ (based on the weight of CoFe_2_O_4_ and 1.3 mole of Al insertion) are obtained.

**Figure 6:**
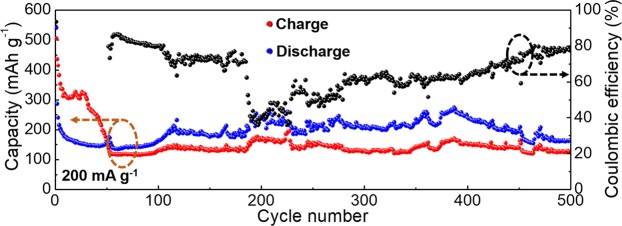
Repeated charge/discharge cycling measurement for the Co_3_O_4_@rGO at 200 mA g^−1^.

Prior to measuring the capacities of the innovative cathode materials, we test the capacity of naked rGO separately. As depicted in Supplementary Fig. S11, negligibly small capacity values for naked rGO are obtained, ensuring the capacities of the metal-oxide NPs@rGO samples in the subsequent measurements originate from the metal-oxide active materials. Surprisingly, unprecedented high capacity values for Co_3_O_4_@rGO are achieved at a current density of 200 mA g^−1^ (Fig. 6), which should be a favourable breakthrough in the attempt of using metal oxides with heavy electronegative O ions as cathode materials for AIBs. After the initial activation phase, an AIB with the Co_3_O_4_@rGO cathode material proceeds for 500 cycles with a retained discharge capacity of 168 mAh g^−1^ and Coulombic efficiency of 76% (Fig. 6). Notably, this result is quite superior to that of the previously reported CuO microspheres and V_2_O_5_ cathode materials in terms of both capacities and lifespan^17,19^. It should be considered that the Al foil can be directly inserted in an anode side without safety concerns, which is quite different from the conventional Li-ion batteries with a requirement of additional consideration for alternatives to Li in an anode side. However, The Coulombic efficiencies increase from 60% to 80% gradually. This relatively low values suggest remain efforts after the primary demonstration for high potentials of metal oxides as cathode materials for rechargeable aluminum-ion batteries in this report.

**Figure 7:**
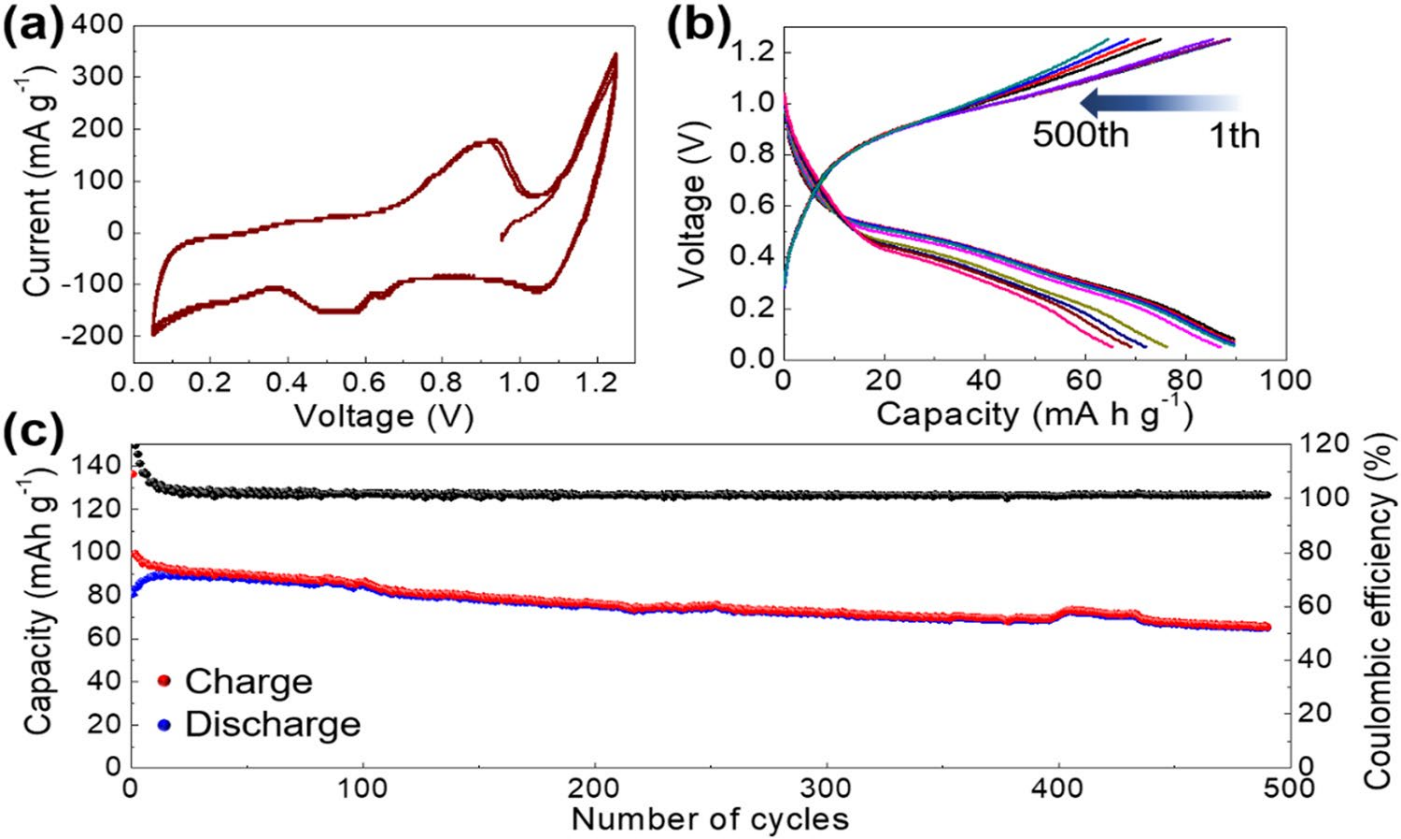
(**a**) CV curve of the CoFe_2_O_4_@rGO measured within a narrowed potential window of 0.05–1.2 V vs. AlCl_4_^−^/Al. (**b**) Representative charge/discharge voltage profiles and (**c**) capacity values of the CoFe_2_O_4_@rGO for continuous charge/discharge at 1,000 mA g^−1^.

Contrary to the Co_3_O_4_@rGO, the Fe_2_O_3_@rGO displays inferior capacities of 29 mAh g^−1^ with a tolerable lifetime (Supplementary Fig. S12a). In addition, CoFe_2_O_4_@rGO shows a high initial discharge capacity of 407 mAh g^−1^ maximum, similar to the Co_3_O_4_@rGO (Supplementary Fig. S12b). However, a rapid decay of capacities for the CoFe_2_O_4_@rGO demonstrates the undesirable electrochemical property. A rational assumption can be due to the deep charge/discharge corresponding to the wide potential range of 0.05–2.2 V vs. AlCl_4_^−^/Al, which may result in a degradation of polymetallic oxide in CoFe_2_O_4_@rGO. We, thus, narrow the potential window from the original range of 0.05–2.2 to 0.05–1.2 V vs. AlCl_4_^−^/Al for further performance analysis. A pair of obvious redox peaks at ~0.5 (reduction) and 0.9 V vs. AlCl_4_^−^/Al (oxidation) are clearly detected in the overlapped (the 1^st^ and 2^nd^) CV curves for CoFe_2_O_4_@rGO after an activation operation (Fig. 7a). Corresponding redox reactions of the redox peaks are pursued following the same method except that the potential window ranges from 0.05 to 1.2 V vs. AlCl_4_^−^/Al without covering the electrochemical reactions at 1.8/2.0 V vs. AlCl_4_^−^/Al for CoFe_2_O_4_@rGO (Supplementary Eqs S11 and S12).

A further long-term charge/discharge demonstration is performed for CoFe_2_O_4_@rGO within the optimized potential window (0.05–1.2 V vs. AlCl_4_^−^/Al) at a high current density of 1,000 mA g^−1^. A capacity value of 67 mAh g^−1^ at the 500^th^ charge/discharge cycle is preserved, corresponding to a retention rate of 74% in comparison with the initial 91 mAh g^−1^, with a high Coulombic efficiency of 99.6% (Fig. 7c). Notably, voltage profiles during the charge/discharge proceeding are captured and representatively provided in Fig. 7b, where the undisputed charge/discharge plateaus are observed at ~0.8 and 0.4 V vs. AlCl_4_^−^/Al, suggesting that the CoFe_2_O_4_@rGO is more appropriately used within a shallow charge/discharge range and is a potential cathode material for AIBs.

TEM characterization is further conducted for the samples after repeated charge/discharge cycling tests. The nanoparticles are well trapped inside rGO as depicted in Supplementary Fig. S13. However, decreased crystallinities are observed suggesting the insertion/extraction of charges upon discharge/charge operation which is consistent with the obtained *ex-situ* XRD results where no obvious peaks are detected other than Al oxides (Supplementary Fig. 10).

Quantitative characterization for the electrochemical impedance of the CoFe_2_O_4_@rGO cathode material is measured using electrochemical impedance spectroscopy (EIS, Supplementary Fig. S14), depicting a depressed semicircle (charge transfer) connected to an oblique line (mass transfer). The EIS curve is studied by incorporating an equivalent circuit (Supplementary Fig. S14 inset). In which, internal resistance (*R*_s_, ~40 Ω) including electrolyte resistance, electrode resistance, electrode/current collector contact resistance, and current collector resistance is shown. Furthermore, charge transfer resistance (*R*_ct_, ~500 Ω), Warburg impedance (*Z*_w_) representing the ionic diffusion resistance, and double layer capacitance (*C*_1_) at the electrode/electrolyte interface are depicted in the EIS curve.

## Conclusions

In this report, we demonstrate high electrochemical activity of economical metal oxides encapsulated in rGO (Co_3_O_4_@rGO, Fe_2_O_3_@rGO, and CoFe_2_O_4_@rGO) towards Al-ion storage as cathode materials for AIBs. The Co_3_O_4_@rGO displays highly improved electrochemical properties regarding both capacity and lifespan which are superior to the state-of-the-art metal oxide cathode material currently reported by scientific literature. Besides, the CoFe_2_O_4_@rGO exhibits an extremely stable charge/discharge process with a promising Coulombic efficiency of 99.6% after an optimization for the operating voltage range. This report is expected to stimulate further investigation on metal oxides as economical cathode materials for high performance AIBs.

## Methods

### Synthesis procedure

In this report, we synthesized rGO-encapsulated metal-oxide NPs (Co_3_O_4_@rGO, Fe_2_O_3_@rGO, and CoFe_2_O_4_@rGO) by a facile spontaneous self-assembly process. The MOF precursors (Co^2+^-hexacyanocobaltate (II), Fe^2+^-hexacyanoferrate (III), and Fe^2+^-hexacyanocobaltate (III)) were prepared by a co-precipitation method in aqueous solutions. After a sufficient precipitation reaction, the collected products were rinsed with deionized water several times to remove impure ions from the reactants. The well-cleaned powders were dried in a vacuum oven overnight, ready for the next usage. The metal-oxide NPs were obtained by heating the as-prepared MOF precursors at 700 °C for 5 h in air. An aqueous solution comprising of the oxidized metal NPs and polydiallyl dimethyl ammonium chloride (PDDA, 5% by volume) was prepared. The solution was constantly stirred to uniformly disperse the NPs by surficial electrostatic repulsion. After stirring overnight, the excess PDDA was removed by centrifugation, washing, and re-dispersion in another aqueous solution. Afterwards, a diluted graphene suspension (10 g L^−1^) was dropwise added to the metal-oxide NPs suspension under constant stirring. This solution was then constantly stirred for further 24 h. During this period, the metal-oxide NPs would spontaneously attach to and be encapsulated by graphene sheets via electrostatic attraction between the positively charged metal-oxide NPs and the negatively charged graphene sheets. The well-assembled products were filtrated and further annealed at 500 °C in an argon atmosphere for 2 h.

### Characterizations

A structural study was performed via Cu-Kα radiation equipped XRD (D8-Advance) at a fixed incident angle of 2°, XPS (PHI 5000 VersaProbe) with an Al-Kα source (Sigma probe, VG Scientifics), and Raman spectroscopy (inVia Raman Microscope). Morphologies and constitution elements were demonstrated via SEM (SUPRA 55VP), TEM (JEOL JEM-2100F), EDX mapping, and EPMA. Thermal stability of the as-prepared composites was demonstrated via TGA, which was performed under air flow with temperature ranging from room temperature to 700 °C (10 °C min^−1^). The ratios of rGO and metal oxides were determined with XRF (ZSX-PRIMUS) technique.

### Electrochemical property

The as-prepared samples were manually grinded together with super P and polyvinylidene fluoride with a weight ratio of 7:2:1; the mixed powders were then dispersed into a constantly stirred *n*-methyl-2-pyrolidinon solution to prepare a uniform slurry for the subsequent electrochemical characterizations. The well-prepared slurry was casted on an organic polymer current collector coated with Pt for conductivity, followed by drying in a vacuum oven overnight to be ready for the subsequent electrochemical measurements.

Electrochemical properties were characterized in pouch cells where the well-dried electrodes were inserted as the cathodes and Al metal foils (0.5 mm) were used as anodes. Two pieces of glass-fiber (Whatman) papers soaked with 1-ethyl-3-methylimidazolium chloride ([EMIM]Cl)/AlCl_3_ (1/1.3 molar ratio) were inserted between anodes and cathodes for insulation.

An EIS analysis of the CoFe_2_O_4_@rGO cathode material, after a repeated galvanostatic charge/discharge process for 10 cycles at 100 mA g^−1^, was performed by using the Im6ex ZAHNER. A frequency range used was from 10 mHz to 1 MHz, under a constant voltage amplitude of 10 mV.

CV measurements were performed on an electrochemical workstation (WBCS3000, Wonatech, Korea) in potential ranges of 0.05–2.2 and 0.05–1.2 V vs. AlCl_4_^−^/Al at a scan rate of 0.5 mV s^−1^. Galvanostatic charge/discharge cycling measurements were performed within potential windows of 0.05–2.2 and 0.05–1.2 V vs. AlCl_4_^−^/Al at various current densities of 100, 200 and 1,000 mA g^−1^. Unless otherwise noted, all the current densities and specific capacities in this study were calculated based on the weight of the metal-oxide NP active materials.

### *Ex-situ* characterizations

Pouch cells with electrodes discharged to desired voltages were disassembled, followed by rinsing the cathodes with ethanol and drying in an oven for *ex-situ* XRD and EDX characterizations.

## Supplementary information

Metal-organic framework-derived metal oxide nanoparticles@reduced graphene oxide composites as cathode materials for rechargeable aluminium-ion batteries

## Data Availability

The data that support the findings of this study are available from the corresponding authors upon reasonable request.
