# Supplementary material for: Metal-organic framework-derived metal oxide nanoparticles@reduced graphene oxide composites as cathode materials for rechargeable aluminium-ion batteries
Source: Sci Rep. 2019 Sep 24;9:13739. doi: 10.1038/s41598-019-50156-6 (PMC6760151; doi:10.1038/s41598-019-50156-6)
Supplement: Supplementary file 1 — Metal-organic framework-derived metal oxide nanoparticles@reduced graphene oxide composites as cathode materials for rechargeable aluminium-ion batteries [file 41598_2019_50156_MOESM1_ESM.docx]

**Supplementary Information**

**Metal-organic framework-derived metal oxide nanoparticles@reduced graphene oxide composites as cathode materials for rechargeable aluminium-ion batteries**

Kaiqiang Zhang^1,2^, Tae Hyung Lee^1^, Joo Hwan Cha^3^, Ho Won Jang^1^, Ji-Won Choi^2*^, Morteza Mahmoudi^4*^ & Mohammadreza Shokouhimehr^1,4*^

^1^Department of Materials Science and Engineering, Research Institute of Advanced Materials, Seoul National University, Seoul 08826, Republic of Korea.

^2^Electronic Materials Center, Korea Institute of Science and Technology (KIST), Seoul 136-791, Republic of Korea.

^3^Innovative Enterprise Cooperation Center, Korea Institute of Science and Technology (KIST), Seoul, Republic of Korea.

^4^Precision Health Program, Michigan State University, East Lansing, MI 48823, USA.

^*^ Corresponding authors

jwchoi@kist.re.kr (J.-W. Choi), Mahmou22@msu.edu (M. Mahmoudi), mrsh2@snu.ac.kr (M. Shokouhimehr)

**
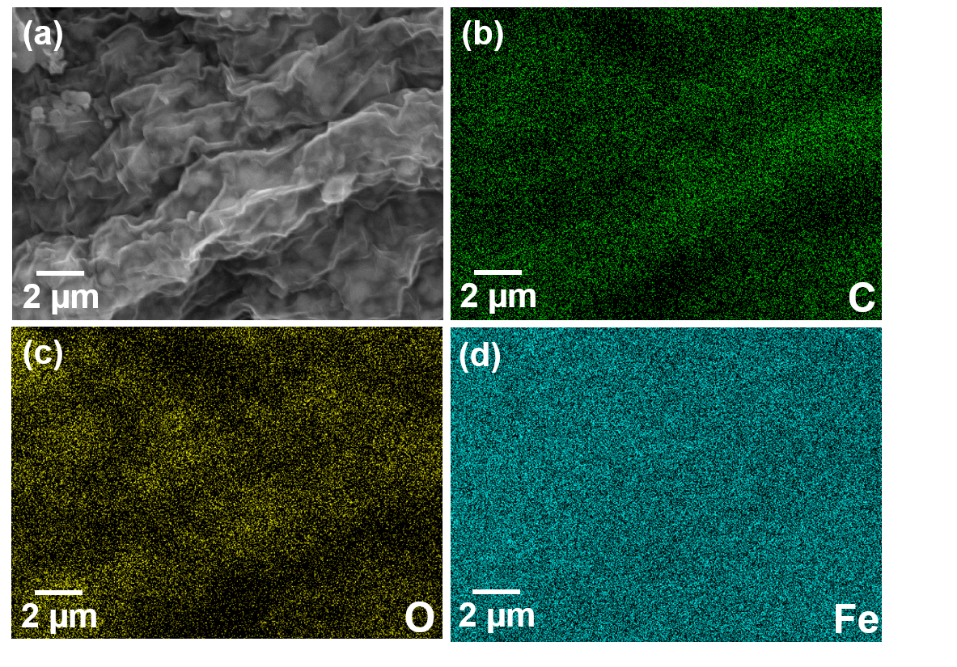
**

**Figure S1.** (**a**) SEM and (**b**–**d**) EDX mapping images of the prepared Fe_2_O_3_@rGO.

**
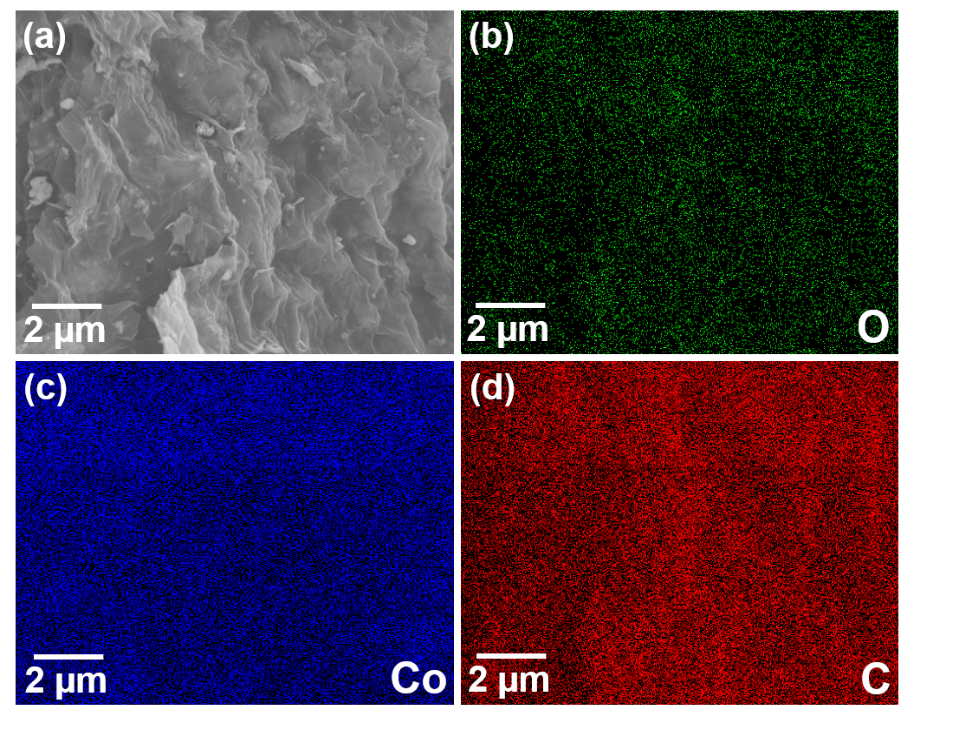
**

**Figure S2.** (**a**) SEM and (**b**–**d**) EDX mapping images of the prepared Co_3_O_4_@rGO.

**
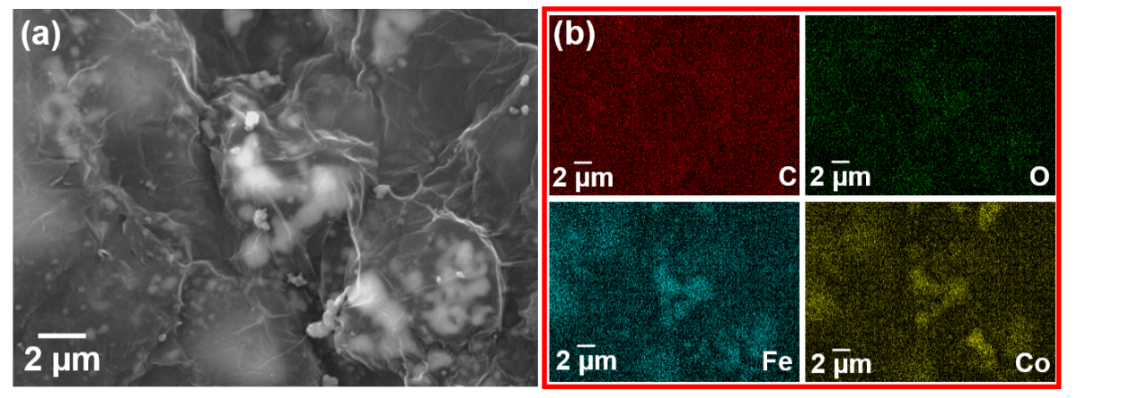
**

**Figure S3.** (**a**) SEM and (**b**) EDX mapping images of the prepared CoFe_2_O_4_@rGO.


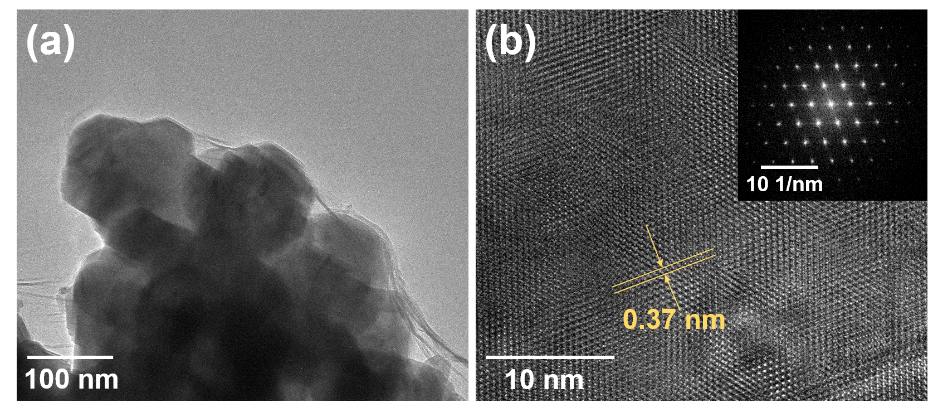


**Figure S4.** (**a**) TEM and (**b**) HRTEM images of the Fe_2_O_3_@rGO sample (inset displays the electron diffraction pattern).

**
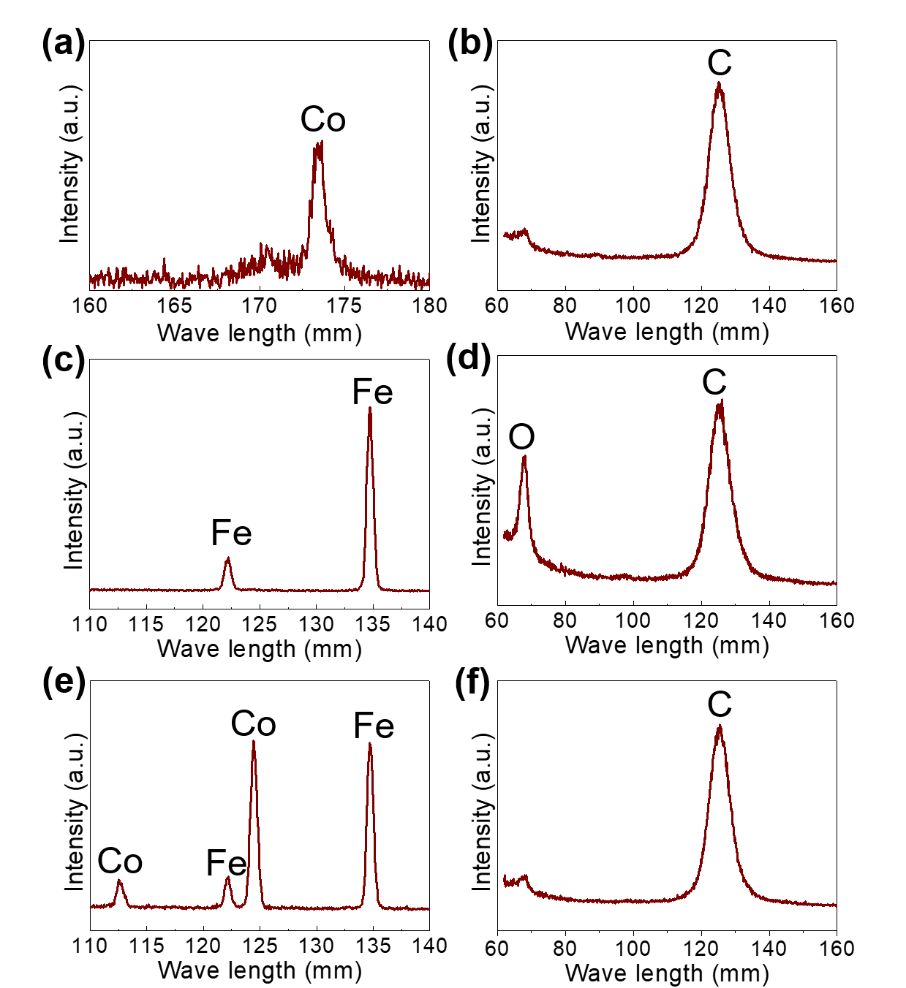
**

**Figure S5.** Electron probe microanalyser spectra of (**a**,**b**) Co_3_O_4_@rGO, (**c**,**d**) Fe_2_O_3_@rGO, and (**e**,**f**) CoFe_2_O_4_@rGO.

**
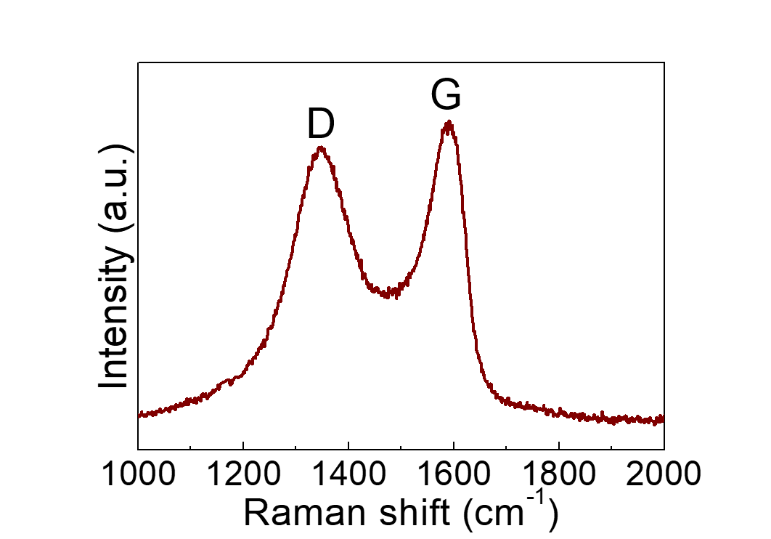
**

**Figure S6.** Raman spectrum of bare rGO.

**
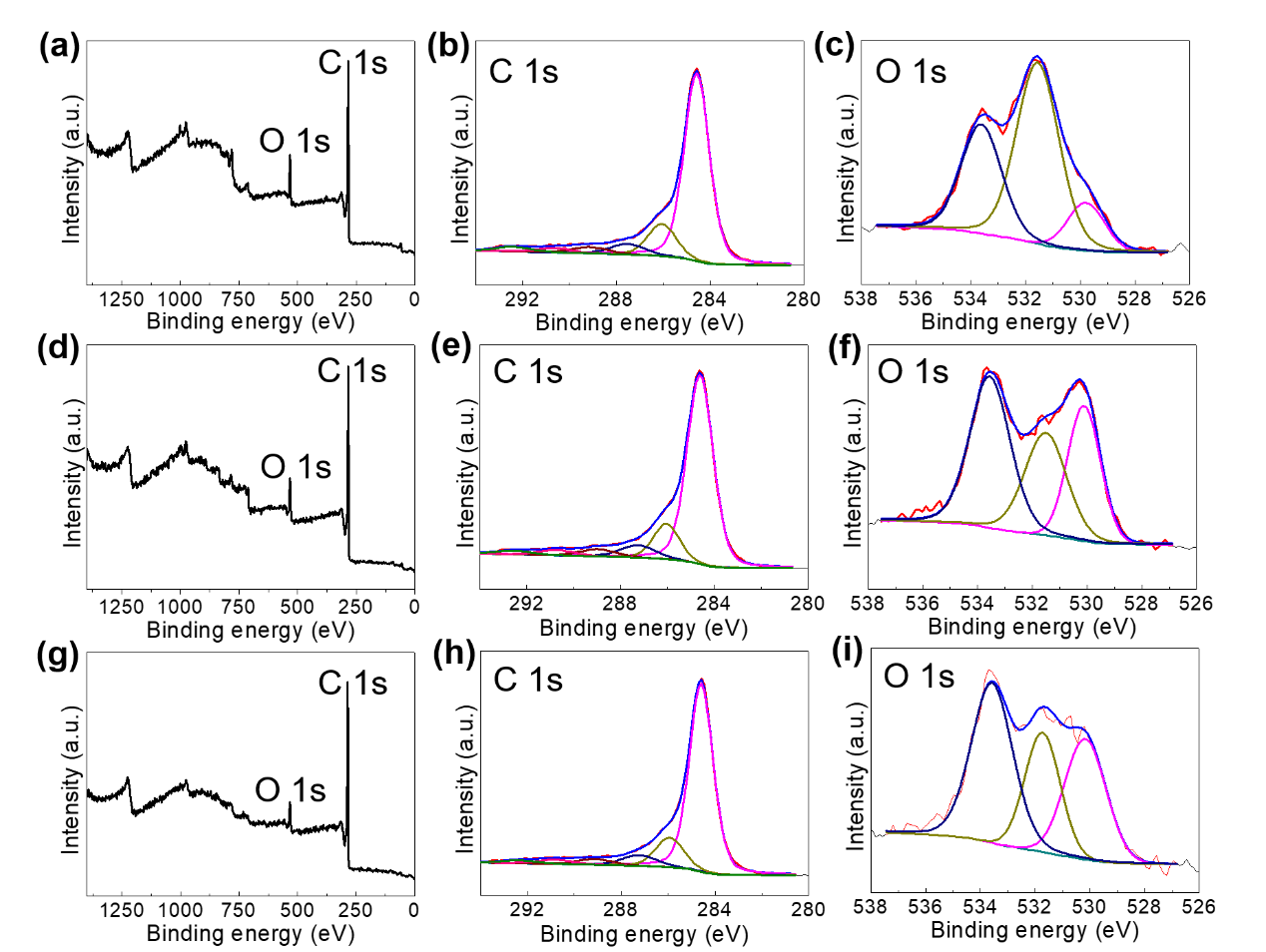
**

**Figure S7.** (**a**,**d**,**g**) Wide survey scan, (**b**,**e**,**h**) C 1s, and (**c**,**f**,**i**) O 1s XPS spectra of the Co_3_O_4_@rGO (up), Fe_2_O_3_@rGO (middle), and CoFe_2_O_4_@rGO (bottom) samples.

**
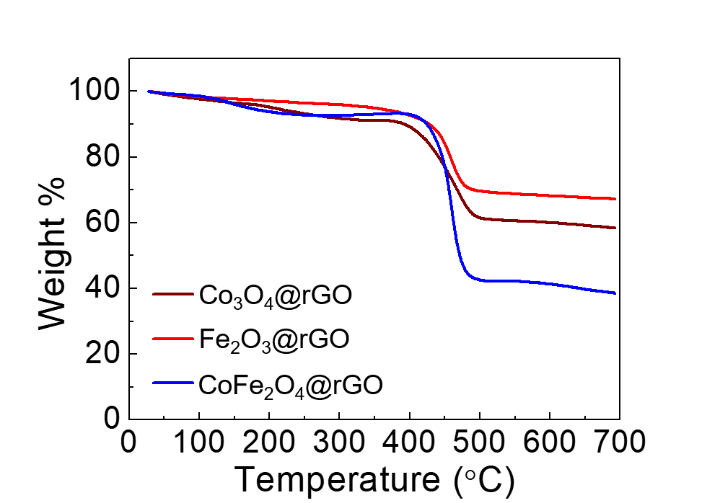
**

**Figure S8.** TGA curves of the as-synthesized Co_3_O_4_@rGO, Fe_2_O_3_@rGO, and CoFe_2_O_4_@rGO.

**
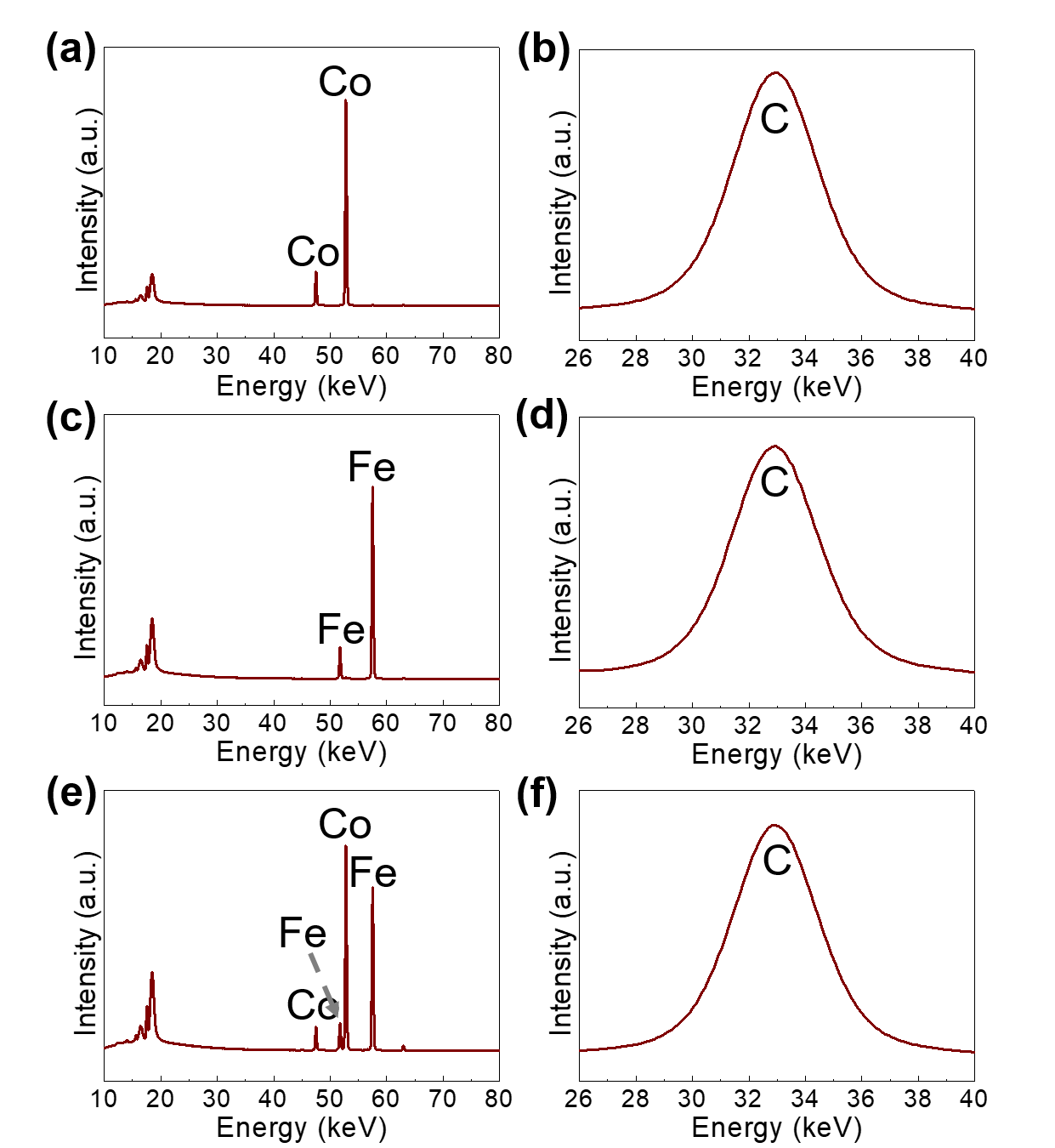
**

**Figure S9.** XRF spectra of the as-synthesized (**a**,**b**) Co_3_O_4_@rGO, (**c**,**d**) Fe_2_O_3_@rGO, and (**e**,**f**) CoFe_2_O_4_@rGO.


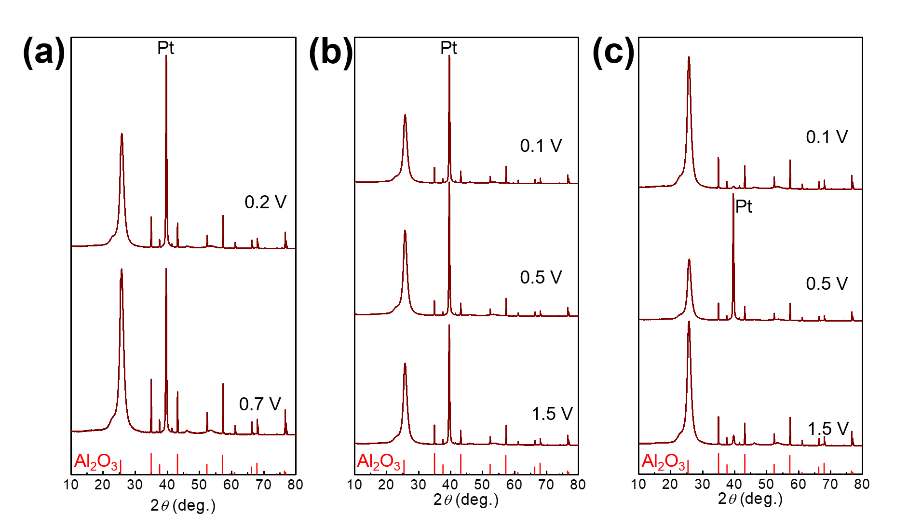


**Figure S10.** XRD spectra of (**a**) Co_3_O_4_@rGO, (**b**) Fe_2_O_3_@rGO, and (**c**) CoFe_2_O_4_@rGO discharged to various potential statuses.

**
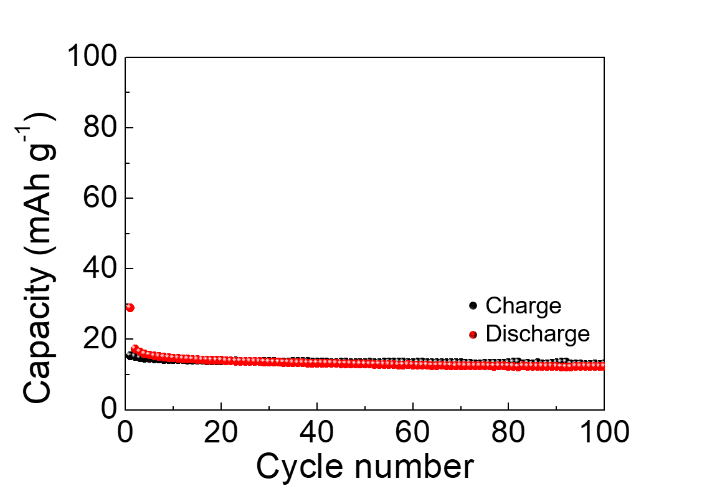
**

**Figure S11.** Consecutive charge/discharge cycling test for naked rGO.

**
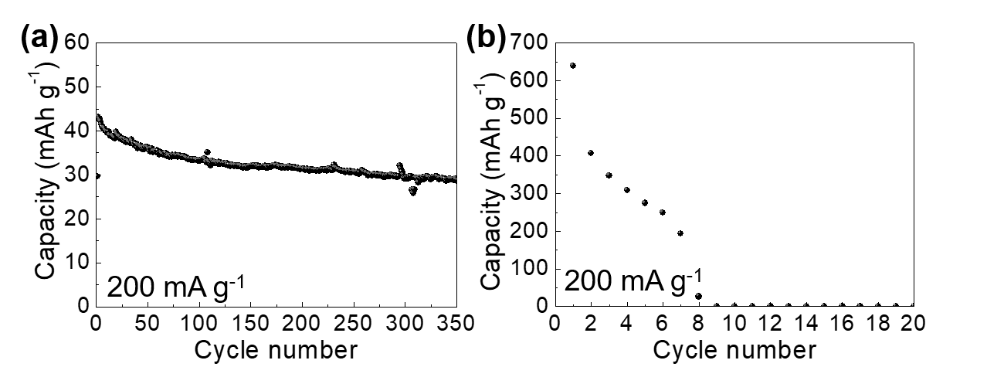
**

**Figure S12.** Repeated charge/discharge measurements for (**a**) Fe_2_O_3_@rGO and (**b**) CoFe_2_O_4_@rGO.


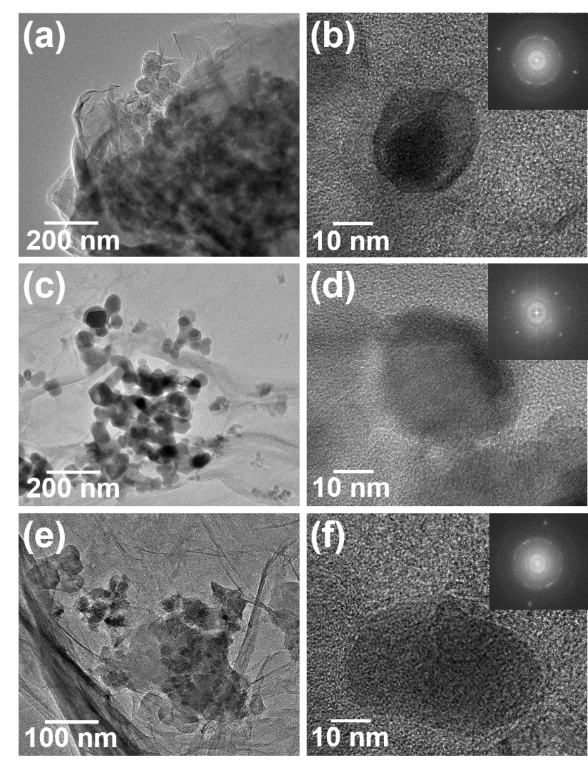


**Figure S13.** TEM images of the (**a**,**b**) Co_3_O_4_@rGO, (**c**,**d**) Fe_2_O_3_@rGO, and (**e**,**f**) CoFe_2_O_3_@rGO samples after repeated charge/discharge cycling characterizations.


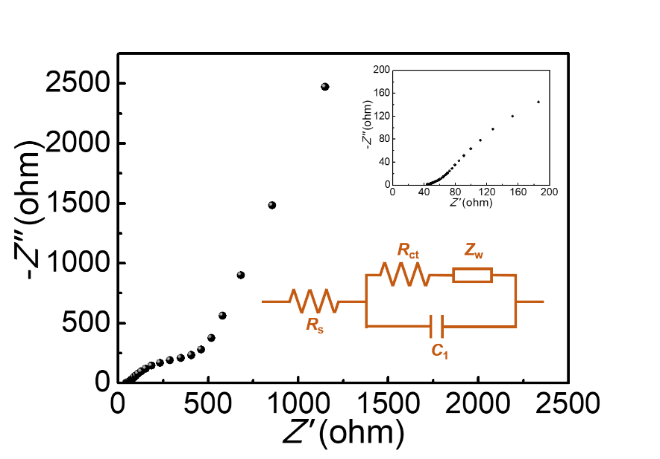


**Figure S14.** EIS curve of the CoFe_2_O_4_@rGO.

**Table S1.** XRF measurement results for the elemental ratios for each material.

|  | Co_3_O_4_@rGO | Fe_2_O_3_@rGO | CoFe_2_O_4_@rGO |
| --- | --- | --- | --- |
| C | 51 | 38 | 60 |
| Metal oxide | 49 | 62 | 40 |

**Table S2.** EDX mapping results for the Co_3_O_4_@rGO discharged to various states.

| **Element** | **At.%** | |
| --- | --- | --- |
|  | **0.2 V** | **0.7 V** |
| C | 73.27 | 73.00 |
| O | 10.10 | 12.97 |
| Al | 02.83 | 01.92 |
| Co | 12.55 | 10.98 |

**Table S3.** EDX mapping results for the Fe_2_O_3_@rGO discharged to various states.

| **Element** | **At.%** | | |
| --- | --- | --- | --- |
|  | **0.1 V** | **0.5 V** | **1.5 V** |
| C | 83.03 | 76.17 | 78.71 |
| O | 16.01 | 07.94 | 06.26 |
| Al | 00.40 | 01.86 | 01.28 |
| Fe | 00.55 | 12.21 | 12.12 |

**Table S4.** EDX mapping results for the CoFe_2_O_4_@rGO discharged to various states.

| **Element** | **At.%** | | |
| --- | --- | --- | --- |
|  | **0.1 V** | **0.5 V** | **1.5 V** |
| C | 70.01 | 72.32 | 70.28 |
| O | 17.70 | 17.05 | 13.59 |
| Al | 02.48 | 01.43 | 00.84 |
| Fe | 03.93 | 03.70 | 06.73 |
| Co | 04.74 | 04.44 | 07.52 |

**Purposed electrochemical reactions:**

**Anode**

Discharge: Al → 3e^-^ + Al^3+^ S1

Charge: 3e^-^ + Al^3+^ → Al S2

**Cathode**

**Co_3_O_4_@rGO**

Discharge to 0.7 V vs. AlCl_4_^-^/Al:

Co_3_O_4_ + 0.52Al^3+^ + 1.56e^-^ → Al_0.52_Co_3_O_4_ S3

Discharge from 0.7 to 0.2 V vs. AlCl_4_^-^/Al:

Al_0.52_Co_3_O_4_ + 0.16Al^3+^ + 0.48e^-^ → Al_0.68_Co_3_O_4_ S4

**Fe_2_O_3_@rGO**

Discharge to 1.5 V vs. AlCl_4_^-^/Al:

Fe_2_O_3_ + 0.21Al^3+^ + 0.63e^-^ → Al_0.21_Fe_2_O_3_ S5

Discharge from 1.5 to 0.5 V vs. AlCl_4_^-^/Al:

Al_0.21_Fe_2_O_3_ + 0.09Al^3+^ + 0.27e^-^ → Al_0.3_Fe_2_O_3_ S6

Discharge from 0.5 to 0.1 V vs. AlCl_4_^-^/Al:

Al_0.3_Fe_2_O_3_ + 1.15Al^3+^ + 3.45e^-^ → Al_1.45_Fe_2_O_3_ S7

**CoFe_2_O_4_@rGO**

Discharge to 1.5 V vs. AlCl_4_^-^/Al:

CoFe_2_O_4_ + 0.25Al^3+^ + 0.75e^-^ → Al_0.25_CoFe_2_O_4_ S8

Discharge from 1.5 to 0.5 V vs. AlCl_4_^-^/Al:

Al_0.25_CoFe_2_O_4_ + 0.52Al^3+^ + 1.56e^-^ → Al_0.77_CoFe_2_O_4_ S9

Discharge from 0.5 to 0.1 V vs. AlCl_4_^-^/Al:

Al_0.77_CoFe_2_O_4_ + 0.5Al^3+^ + 1.5e^-^ → Al_1.27_CoFe_2_O_4_ S10

**CoFe_2_O_4_@rGO**

Discharge from 1.2 to 0.05 V vs. AlCl_4_^-^/Al:

CoFe_2_O_4_ + 0.52Al^3+^ + 1.56e^-^ → Al_0.52_Fe_2_O_4_ S11

Al_0.52_CoFe_2_O_4_ + 0.5Al^3+^ + 1.5e^-^ → Al_1.02_Fe_2_O_4_ S12

Electrochemical reactions for the charge process proceed in an opposite approach.
